# A study on the use of acupoint catgut embedding in the treatment of pre-diabetes: a meta-analysis and data mining approach

**DOI:** 10.3389/fpubh.2023.1282720

**Published:** 2023-12-07

**Authors:** Yunfeng Yu, Xuan Xu, Danni Tan, Yuman Yin, Xinyu Yang, Rong Yu

**Affiliations:** ^1^Endocrinology Department, The First Hospital of Hunan University of Chinese Medicine, Changsha, Hunan, China; ^2^College of Chinese Medicine, Hunan University of Chinese Medicine, Changsha, Hunan, China

**Keywords:** pre-diabetes, meta-analysis, data mining, acupoint catgut embedding, acupoint, Chinese medicine

## Abstract

**Objective:**

The efficacy of acupoint catgut embedding (ACE) for the treatment of pre-diabetes remains controversial. Therefore, this study investigated the clinical efficacy and acupoint selection in ACE for the treatment of pre-diabetes.

**Methods:**

Eight common databases were searched for relevant literature on ACE for pre-diabetes. Meta-analysis was used to evaluate its efficacy and safety, and data mining was used to explore the protocol for acupoint selection.

**Results:**

The meta-analysis revealed that compared with conventional treatment alone, conventional treatment combined with ACE reduced the levels of glycated hemoglobin A1c [mean difference (MD) −0.45, 95% confidence interval (CI) −0.67 to −0.24%, *p* < 0.001], fasting blood glucose (MD −0.61 mmol/L, 95% CI −0.87 to −0.36 mmol/L, *p* < 0.001), 2-h postprandial glucose (MD −0.77 mmol/L, 95% CI −0.98 to −0.55 mmol/L, *p* < 0.001), total cholesterol (MD −0.37 mmol/L, 95% CI −0.74 to 0.00 mmol/L, *p* = 0.049), triglyceride (MD −0.49 mmol/L, 95% CI −0.77 to −0.20 mmol/L, *p* < 0.001) and low-density lipoprotein cholesterol (MD −0.23 mmol/L, 95% CI −0.33 to −0.12 mmol/L, *p* < 0.001), and increased high-density lipoprotein cholesterol levels (MD 0.16 mmol/L, 95% CI 0.05 to 0.27 mmol/L, *p* = 0.004), whereas changes in the body mass index and the adverse event rates were comparable between groups. Data mining revealed that Pishu (BL20), Weiwanxiashu (EX-B3), Zusanli (ST36), Shenshu (BL23), Sanyinjiao (SP6), Weishu (BL21), and Taixi (KI3) were the core acupoints used in ACE for pre-diabetes.

**Conclusion:**

ACE can effectively improve blood glucose and lipid levels in pre-diabetes patients and has a good safety profile. ACE consisting of Pishu (BL20), Weiwanxiashu (EX-B3), Zusanli (ST36), Shenshu (BL23), Sanyinjiao (SP6), Weishu (BL21), and Taixi (KI3), is a promising complementary strategy for the treatment of pre-diabetes.

## Introduction

Pre-diabetes is a high-risk state in which blood glucose is between normal and diabetes thresholds, including impaired fasting glucose (IFG) and impaired glucose tolerance (IGT) (1). IFG is defined as a fasting glucose of 6.1–6.9 mmol/L, and IGT is defined as a 2-h postprandial glucose (2 h PG) of 7.8–11.0 mmol/L on the oral glucose tolerance test (2). Approximately 14.7% (750 million) of the global population currently have IFG and IGT, and the disease burden is expected to increase in the next 20 years (3). In the United States, approximately 10% of people with pre-diabetes develop diabetes each year, and they are at a significantly increased risk of complications such as cardiovascular disease and death (4). Lifestyle interventions, such as dietary control and exercise, are first-line treatment strategies for pre-diabetes, but many pre-diabetes patients have difficulty adhering to these strategies (5). Although some drugs, such as metformin, are used in the treatment of pre-diabetes, insulin resistance or impaired secretion did not change when patients stopped taking the drug, and the incidence of diabetes at 10 years was similar between patients who continued to take metformin and those who did not take the drug (6, 7). Therefore, novel therapeutic strategies require exploration to lower blood glucose levels and improve insulin resistance in pre-diabetes patients.

Acupuncture is a traditional Chinese medical treatment that has demonstrated good efficacy in lowering blood glucose levels in diabetes patients (8), reducing weight in obese patients (9), and improving the prognosis of other diseases (10). Acupoint catgut embedding (ACE) is a special type of acupuncture therapy in which absorbable surgical sutures are embedded into acupoints. ACE is used to treat diseases and strengthen the body by continuously stimulating the acupoints (11). ACE has the characteristics of minimal trauma, strong stimulation, long-lasting effects, and a low incidence of adverse reactions, and has been widely used in China for the treatment of obesity and other diseases (12). ACE may solve the difficulty faced by patients in adhering to lifestyle interventions. According to previous reports, ACE regulates glucose metabolism, improves islet function, and reduces visceral fat accumulation, and reduces the risk of diabetes and its complications (13, 14). However, no meta-analyses or data mining have been conducted to assess the effectiveness of ACE for pre-diabetes, and its efficacy and acupoint selection protocol remain unclear. Therefore, this study aimed to evaluate the benefits and risks of ACE in the treatment of pre-diabetes through a meta-analysis and exploring acupoint selection of ACE in the treatment of pre-diabetes through data mining.

## Methods

A systematic review and meta-analysis was conducted according to the methodology specified in the Preferred Reporting Items for Systematic Reviews and Meta-Analyses (PRISMA) (15) and was registered in PROSPERO (CRD42023456950).

### Literature search

Four English databases (Embase, PubMed, the Cochrane Library, and the Web of Science) and four Chinese databases (China National Knowledge Infrastructure, China Biology Medicine, VIP, and Wanfang) were searched for articles on ACE for pre-diabetes from their inception up to June 2023. The subject terms of the study were “acupoint catgut embedding” and “pre-diabetes,” and free terms were obtained using VIP and medical subject headings (MeSH). The subject and free terms were combined for the search.

### Inclusion and exclusion criteria

The inclusion criteria were: (i) Randomized controlled trials; (ii) Inclusion of pre-diabetes patients (16); (iii) Participants in the control group received conventional treatment, and those in the experimental group received ACE and conventional treatment. (iv) Efficacy endpoints were blood glucose [glycated hemoglobin A1c (HbA1c), fasting blood glucose (FBG), 2 h PG], blood lipid [total cholesterol (TC), triglyceride (TG), low-density lipoprotein cholesterol (LDL-C), and high-density lipoprotein cholesterol (HDL-C)], body mass index (BMI) and the clinical effective rate, and safety endpoints were adverse events.

The exclusion criteria were: (i) Repeated publications; (ii) Incomplete research data; (iii) Data not available.

### Literature screening, data statistics, and risk of bias

First, the basic literature was imported into the Reference Aid for Medicine, and the included literature was obtained by layer-by-layer screening according to the inclusion and exclusion criteria. Second, the included studies were categorized and organized, and the baseline information of each study was entered into a basic characteristics table. The risk of bias was assessed using the Cochrane risk-of-bias assessment tool. All tasks were performed independently by Yunfeng Yu and Xuan Xu and any disagreements were adjudicated by Danni Tan.

### Data analysis

Meta-analysis was performed using Revman5.3, with risk ratio (RR) and 95% confidence interval (CI) as effect sizes for dichotomous variables and mean difference (MD) and 95% CI for continuous variables. Heterogeneity was analyzed using the *I*^2^-test, and a fixed-effects model was used when *I*^2^ was <50%; otherwise, a random-effects model was used. Sensitivity analyses were performed on the indicators with significant heterogeneity to determine whether the results were robust. Publication bias was assessed by performing a Harbord regression using Stata15.0, and the absence of publication bias was demonstrated if *p* > 0.1. SPSS Modeler 18.0 was used to perform frequency and association rule analyses. The association rule analysis was conducted using the *a priori* model with the settings of support ≥30%, confidence ≥100%, and benefits ≥1.0, to obtain the core combination of acupoints for pre-diabetes by ACE.

## Results

### Literature screening

**Figure 1:**
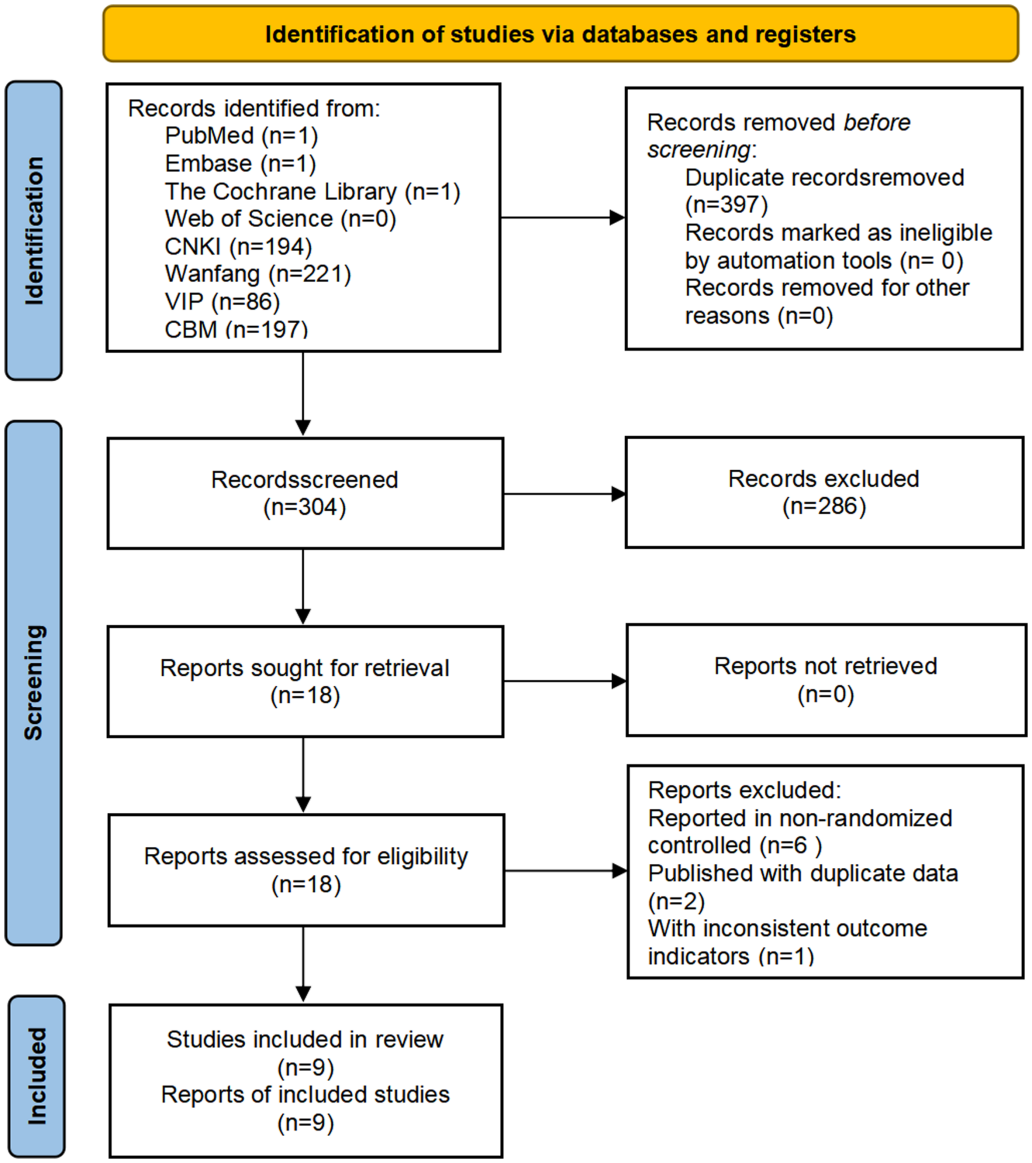
Study flow diagram.

The search yielded 701 relevant studies, and nine relevant studies were included after systematic screening (17–25) (Figure 1).

### Basic characteristics of included studies

The nine included studies (17–25) were all conducted in China and had a total sample size of 868 patients, of whom 434 received conventional treatment and 434 received ACE combined with conventional treatment (Table 1).

**Table 1:** Basic characteristics of inclusion in the study.

Author name	Patient number	Number randomized	Male (%)	Age (years)	Disease duration (months)	Intervention	Treatment duration (weeks)
Wang Y 2021	60	30	50.0	53.9	/	Acupoint catgut embedding q2w health education diet control physical exercise	8
		30	60.0	51.1	/	Health education diet control physical exercise	8
Wang YJ 2015	240	120	55.8	43.0	/	Acupoint catgut embedding q10d diet control physical exercise	12
		120	54.2	52.0	/	Diet control physical exercise	12
Xue N 2017	86	43	41.9	52.0	81.6	Acupoint catgut embedding q15d health education diet control physical exercise	12
		43	46.5	53.0	79.8	Health education diet control physical exercise	12
Yang LX 2022	60	30	56.0	56.0	2.3	Acupoint catgut embedding q15d diet control physical exercise	12
		30	50.0	55.7	2.6	Diet control physical exercise	12
Zhang KY 2021	60	30	53.3	38.9	/	Acupoint catgut embedding q2w metformin 0.5 g tid health education diet control physical exercise	12
		30	56.7	36.6	/	Metformin 0.5 g tid health education diet control physical exercise	12
Zhao BT 2020	74	36	50.0	36.6	11.4	Acupoint catgut embedding q15d health education diet control physical exercise	20
		38	47.4	37.3	11.2	Health education diet control physical exercise	20
Zhao N 2016	70	35	/	46.9	/	Acupoint catgut embedding q10d diet control physical exercise	12
		35	/	46.9	/	Diet control physical exercise	12
Qu FZ 2016	62	31	25.8	47.1	/	Acupoint catgut embedding q4w health education diet control physical exercise	12
		31	22.6	47.2	/	Health education diet control physical exercise	12
Zhang Y 2019	76	39	37.5	42.3	9.7	Acupoint catgut embedding q20d metformin 0.5 g tid diet control physical exercise	24
		37	35.0	45.0	9.0	Metformin 0.5 g tid diet control physical exercise	24
Zhang Y 2019	80	40	40.0	42.8	7.7	Acupoint catgut embedding q20d diet control physical exercise	24
		40	40.0	43.9	8.7	Diet control physical exercise	24

### Risk of bias assessment

**Figure 2:**
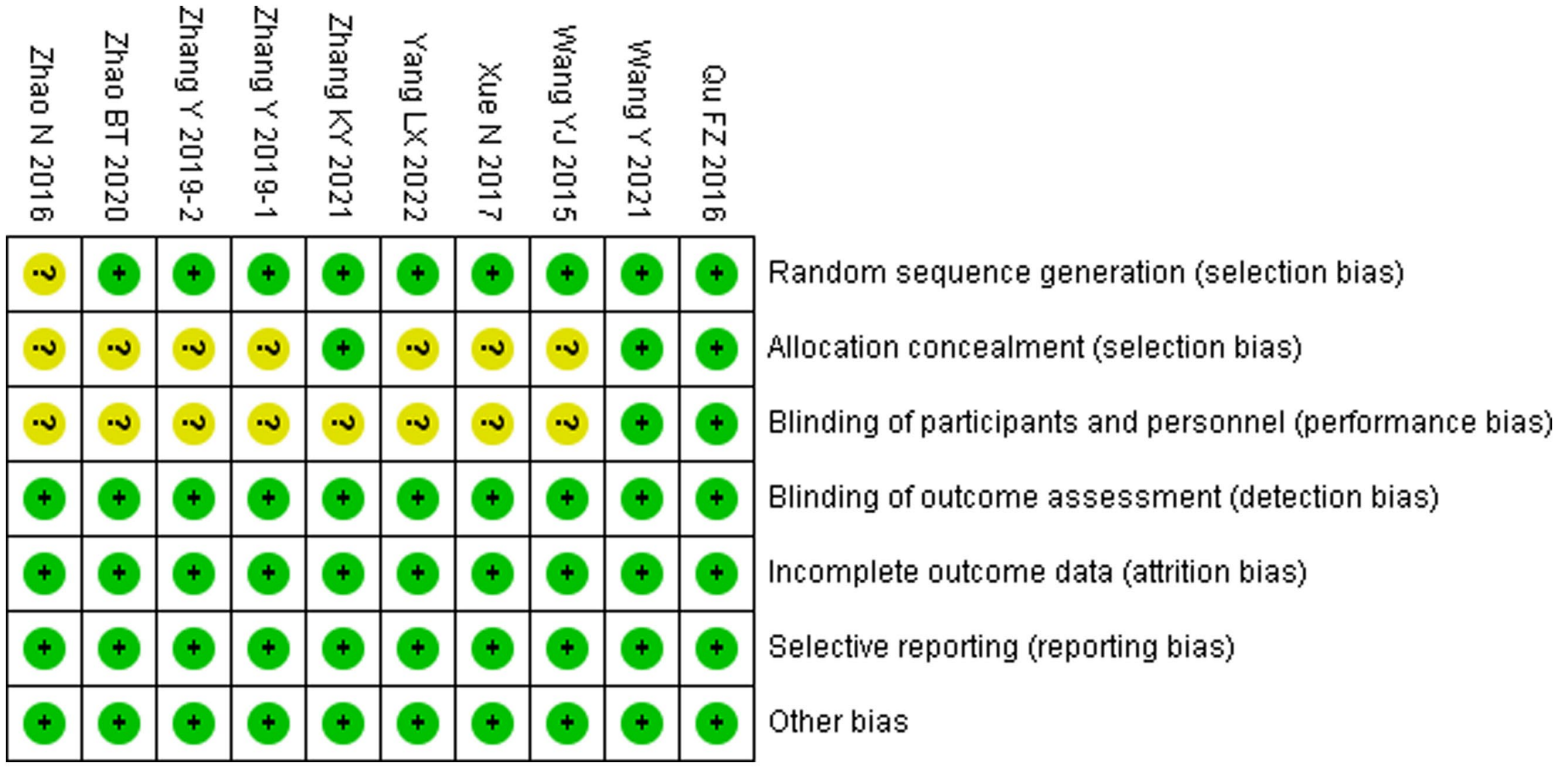
Risk of bias summary.

**Figure 3:**
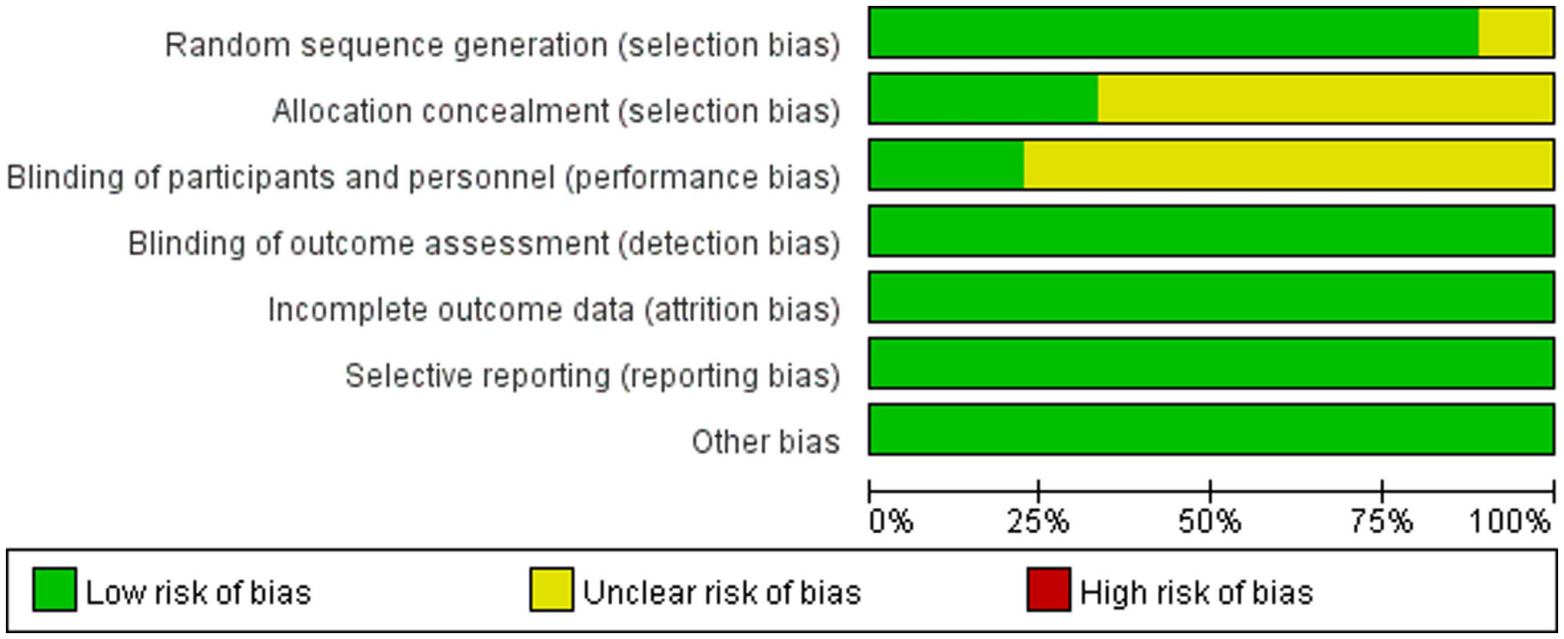
Risk of bias plot.

The risk of bias was unclear for the randomization method in one study, the risk of bias was unclear for allocation concealment in six studies, the risk of bias was unclear for blinding of interventions to patients and participants in seven studies, and the risk of bias was low in the remaining areas (Figures 2, 3).

### Efficacy endpoints

#### Blood glucose

**Figure 4:**
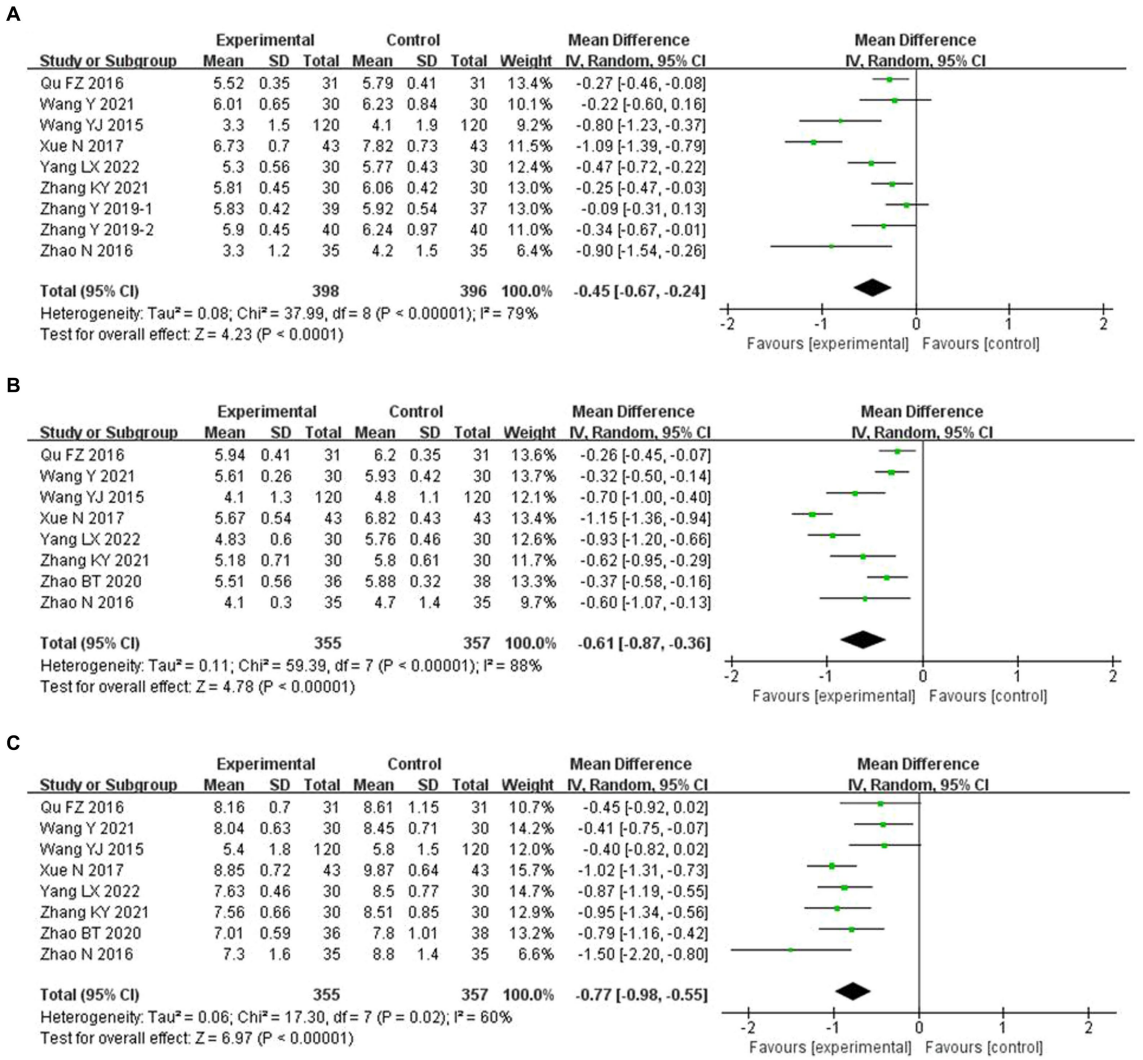
Meta-analysis of blood glucose endpoints in experimental group vs. control group in the treatment of pre-diabetes. **(A)** Meta analysis of HbA1c in experimental group vs. control group in the treatment of pre-diabetes. **(B)** Meta analysis of FBG in experimental group vs. control group in the treatment of pre-diabetes. **(C)** Meta analysis of 2h PG in experimental group vs. control group in the treatment of pre-diabetes.

Compared with conventional treatment, combined ACE and conventional treatment significantly reduced HbA1c (MD −0.45, 95% CI −0.67 to −0.24%, *p* < 0.001), FBG (MD −0.61 mmol/L, 95% CI −0.87 to −0.36 mmol/L, *p* < 0.001) and 2 h PG (MD −0.77 mmol/L, 95% CI −0.98 to −0.55 mmol/L, *p* < 0.001) (Figure 4).

#### Blood lipids

**Figure 5:**
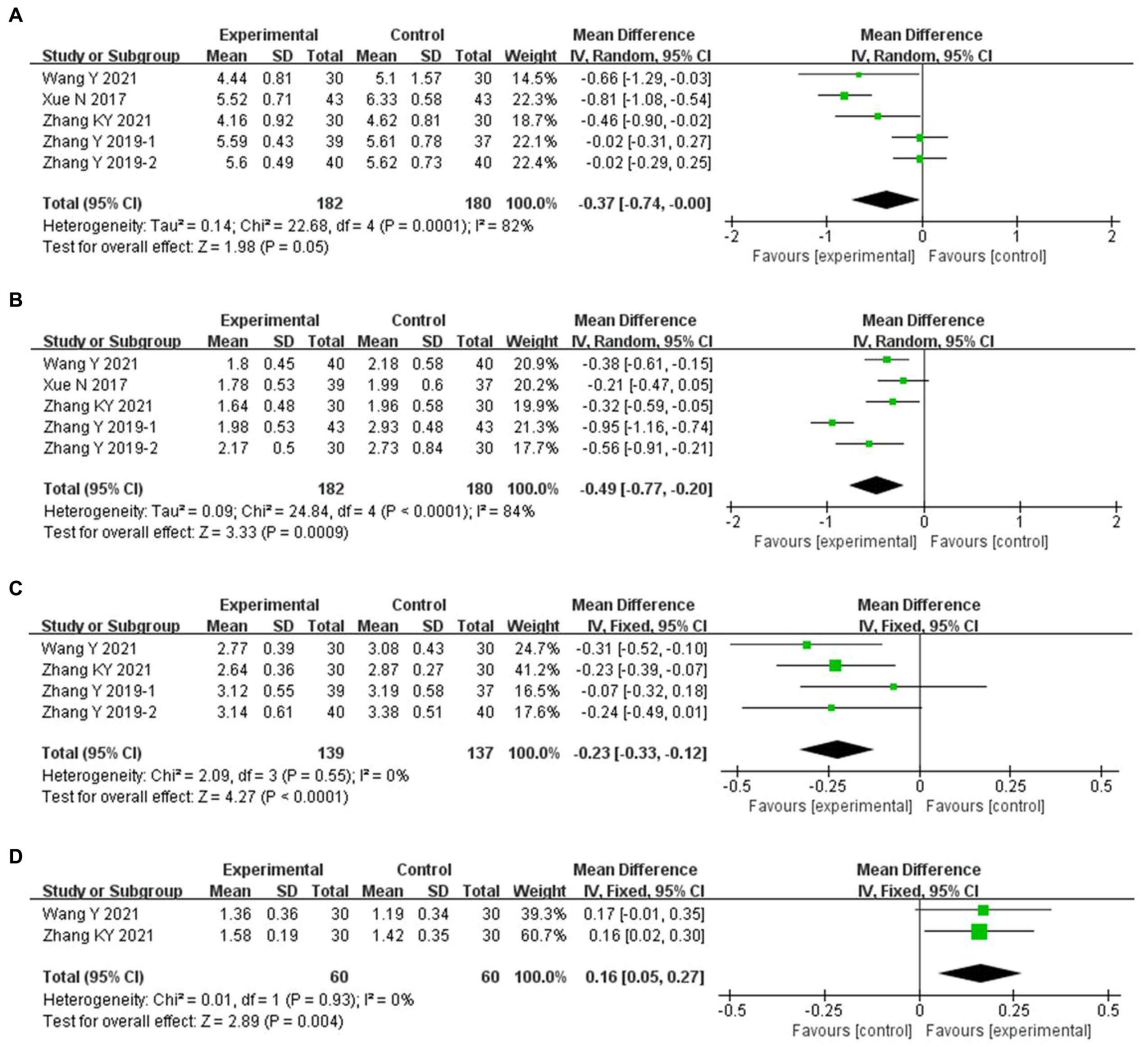
Meta-analysis of blood lipid endpoints in experimental group vs. control group in the treatment of pre-diabetes. **(A)** Meta analysis of TC in experimental group vs. control group in the treatment of pre-diabetes. **(B)** Meta analysis of TG in experimental group vs. control group in the treatment of pre-diabetes. **(C)** Meta analysis of LDL-C in experimental group vs. control group in the treatment of pre-diabetes. **(D)** Meta analysis of HDL-C in experimental group vs. control group in the treatment of pre-diabetes.

Compared with conventional treatment, combined ACE and conventional significantly reduced TC (MD −0.37 mmol/L, 95% CI −0.74 to 0.00 mmol/L, *p* = 0.049), TG (MD −0.49 mmol/L, 95% CI −0.77 to −0.20 mmol/L, *p* < 0.001) and LDL-C (MD −0.23 mmol/L, 95% CI −0.33 to −0.12 mmol/L, *p* < 0.001), and increased HDL-C (MD 0.16 mmol/L, 95% CI 0.05 to 0.27 mmol/L, *p* = 0.004) (Figure 5). The analysis of HDL-C was adjusted using the Hartung-Knapp (HK) method (26). After HK adjustment, HDL-C was still significantly higher in the ACE and conventional treatment group (MD 0.16 mmol/L, 95% CI 0.10 to 0.23 mmol/L, *p* = 0.019), as shown in Supplementary Figure S1.

#### BMI

**Figure 6:**
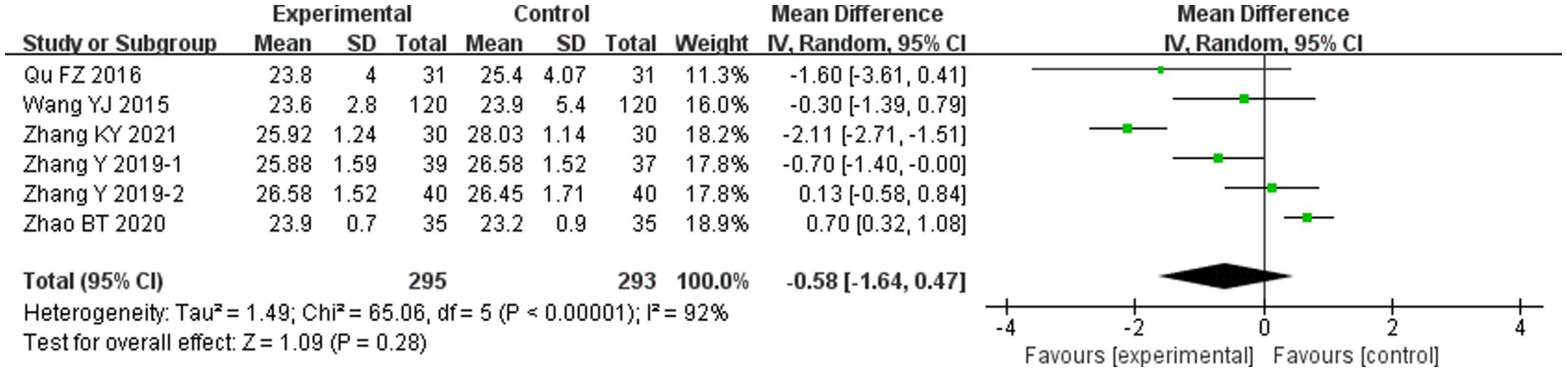
Meta-analysis of BMI endpoints in experimental group vs. control group in the treatment of pre-diabetes.

The combined ACE and conventional treatment groups did not differ significantly in terms of BMI (MD −0.58 kg/m^2^, 95% CI −1.64 to 0.47 kg/m^2^, *p* = 0.28) (Figure 6).

#### Clinical effective rate

**Figure 7:**
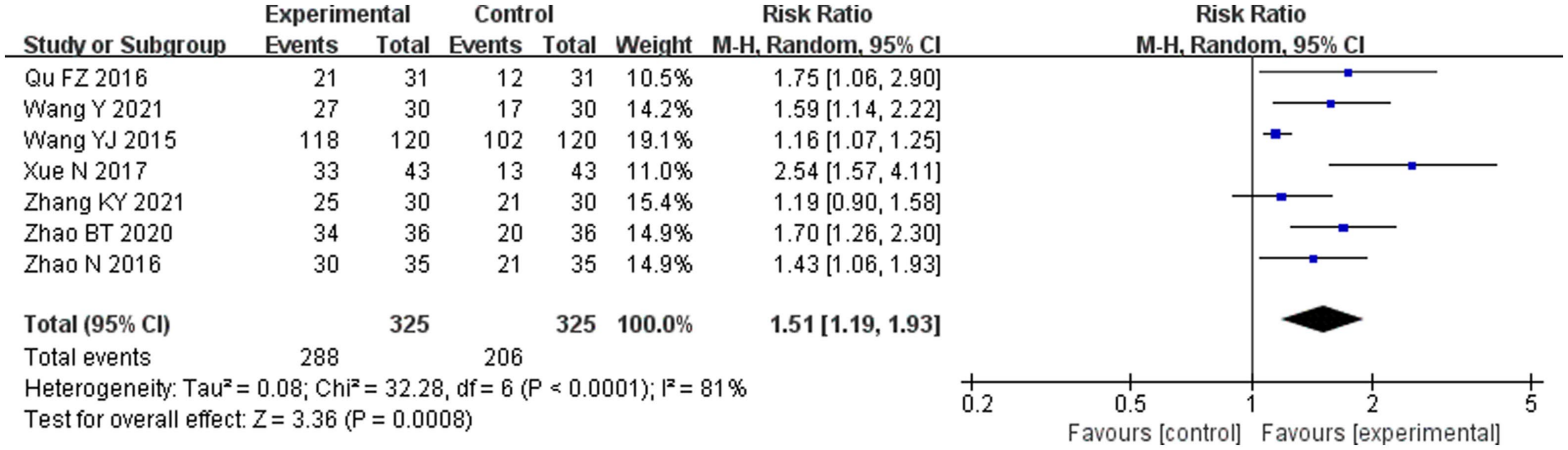
Meta-analysis of the clinical effective rate in experimental group vs. control group in the treatment of pre-diabetes.

Compared with the conventional treatment group, the clinical effective rate in the combined ACE group significantly increased by 51% (RR 1.51, 95% CI 1.19–1.93, *p* < 0.001) (Figure 7).

### Safety endpoints

**Figure 8:**
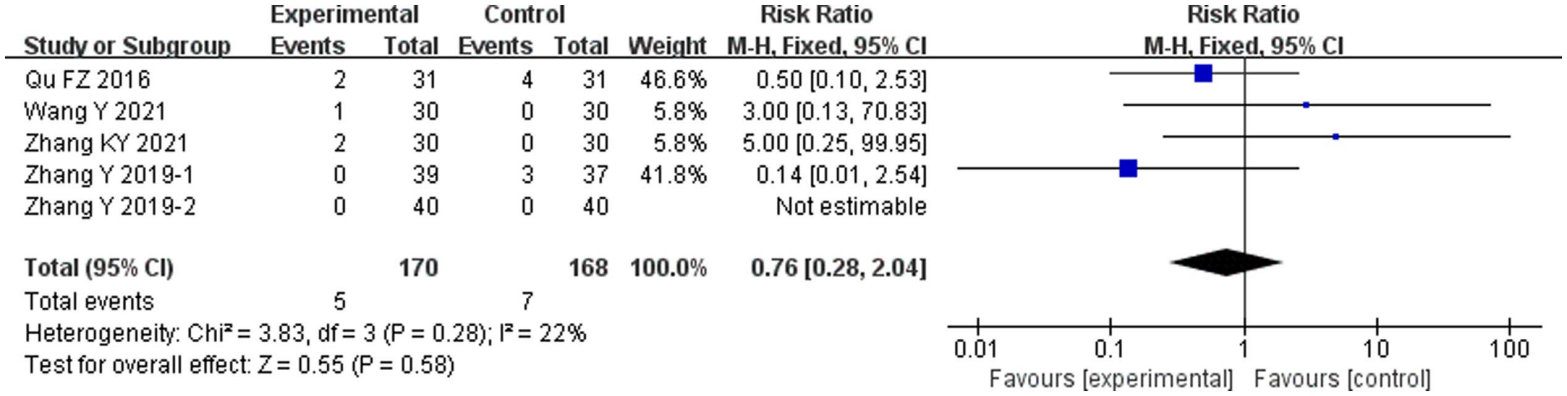
Meta-analysis of the adverse events in experimental group vs. control group in the treatment of pre-diabetes.

Five adverse events were reported in the combined ACE group, including two patients of low-grade fever, two with subcutaneous hematoma, and one with pain. Seven adverse events were reported in the conventional treatment group, including four patients with subcutaneous induration and three with gastrointestinal symptoms. The two groups did not differ significantly in terms of adverse events (RR 0.76, 95% CI 0.28–2.04, *p* = 0.58) (Figure 8).

### Sensitivity analysis

Because there was significant heterogeneity in HbA1c, FBG, 2 h PG, TC, TG, and clinical efficacy rate, sensitivity analysis was used to test the robustness of the combined results. The results indicated that the sensitivity of the combined analysis for HbA1c, FBG, 2 h PG, and TG and the clinically effective rate were low, suggesting that the results were robust. However, the sensitivity of the combined analysis for TC was high, indicating that the results are less credible.

### Subgroup analysis

The subgroup analysis used HbA1c as the outcome and explored the impact of factors such as the frequency of embedding, duration of treatment, and hypoglycemic medication in combination, on the efficacy of ACE for pre-diabetes. The results showed that in terms of frequency of embedding, ACE can effectively reduce HbA1c with the frequency ≤ 15 days (MD −0.60, 95% CI −0.90 to −0.29%, *p* < 0.001) and > 15 days (MD −0.22, 95% CI −0.35 to −0.08%, *p* = 0.002). In terms of course of treatment, ACE for 12 weeks can effectively reduce HbA1c (MD −0.59, 95% CI −0.87 to −0.31%, *p* < 0.001), whereas ACE for 8 weeks (MD −0.22, 95% CI −0.60 to 0.16%, *p* = 0.26) and that for 24 weeks (MD −0.18, 95% CI −0.42 to 0.05%, *p* = 0.13) had no benefit on HbA1c. At the drug level, ACE combined with hypoglycemic drugs (MD −0.17, 95% CI −0.33 to −0.01%, *p* = 0.03) and without hypoglycemic drugs (MD −0.56, 95% CI −0.81 to −0.30%, *p* < 0.001) effectively reduced HbA1c levels (Table 2).

**Table 2:** Subgroup analysis of acupoint catgut embedding in the treatment of pre-diabetes.

Subject	Subgroup	MD (95%CI)	*p-*value
Line change frequency	≤15 days	−0.60 (−0.90 ~ −0.29)	0.0001
	>15 days	−0.22 (−0.35 ~ −0.08)	0.002
Course of treatment	8 weeks	−0.22 (−0.60 ~ 0.16)	0.26
	12 weeks	−0.59 (−0.87 ~ −0.31)	<0.0001
	24 weeks	−0.18 (−0.42 ~ 0.05)	0.13
Hypoglycemic drugs	Combined drugs	−0.17 (−0.33 ~ −0.01)	0.03
	No drugs	−0.56 (−0.81 ~ −0.30)	<0.0001

### Assessment of publication bias

**Figure 9:**
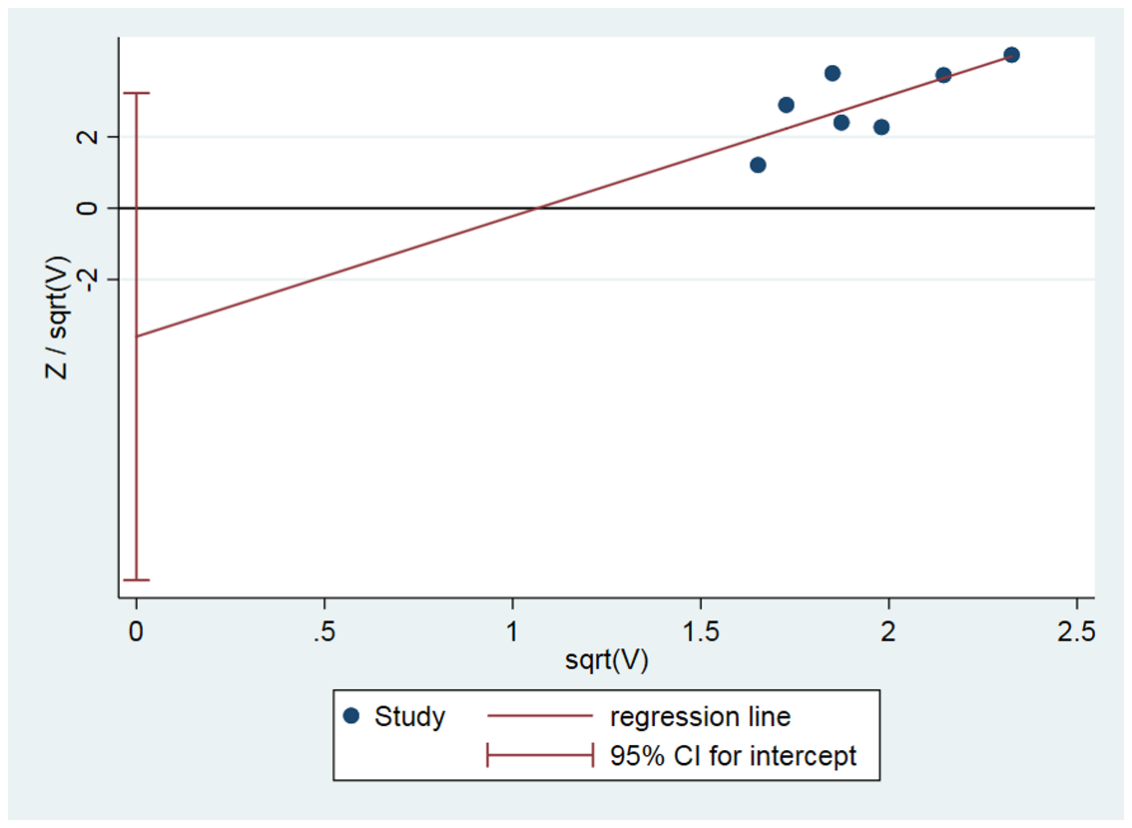
Assessment of publication bias.

The Harbord regression for the clinical efficacy rate was *p* = 0.233, suggesting no significant publication bias (Figure 9).

### Frequency analysis

**Figure 10:**
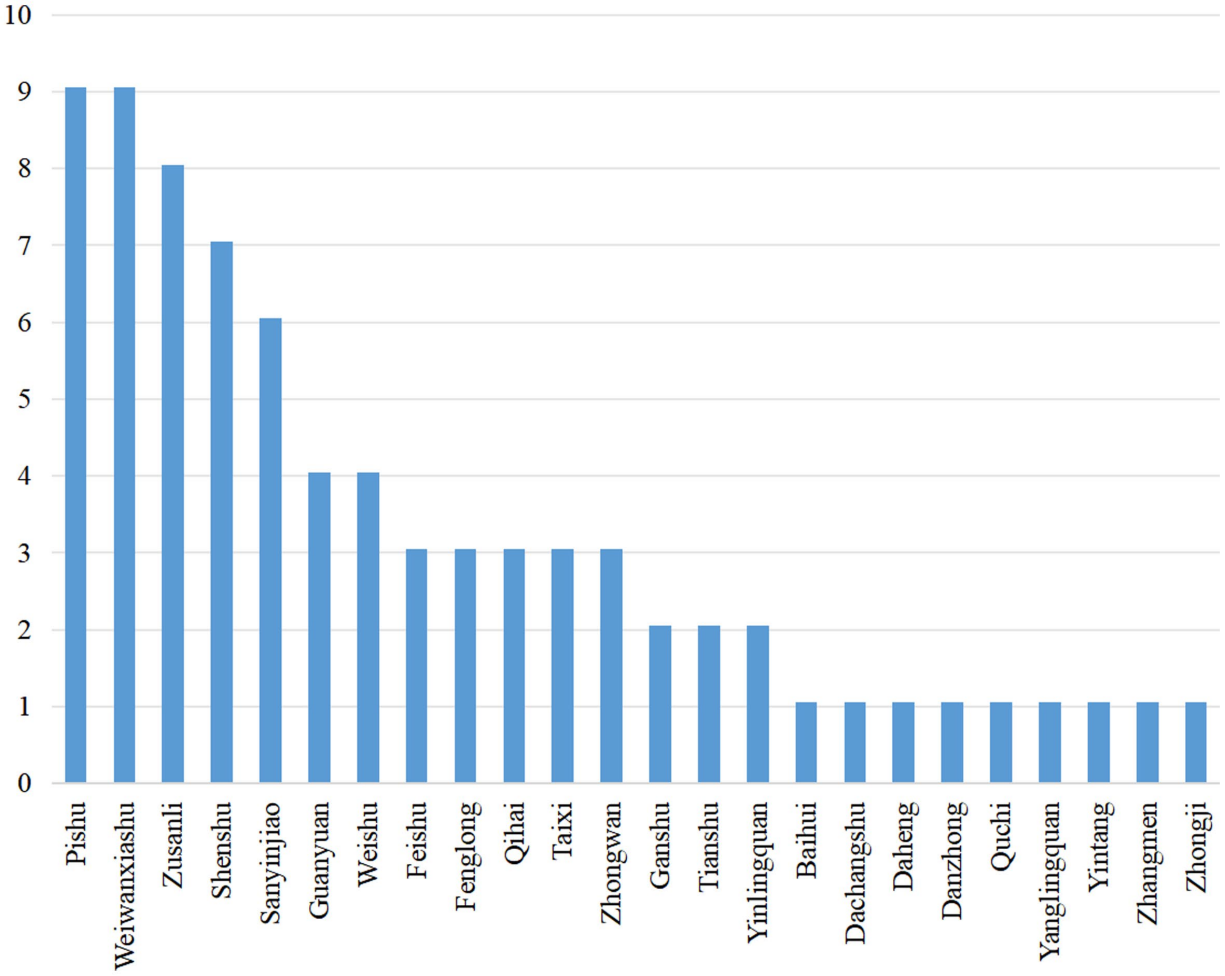
Frequency analysis.

Frequency analysis showed that 9 ACE schemes were included, with a total frequency of 77 times, containing 24 commonly used acupoints for the treatment of pre-diabetes. Among them, the acupoints with ≥50% frequency were Pishu (BL20), Weiwanxiashu (EX-B3), Zusanli (ST36), Shenshu (BL23), and Sanyinjiao (SP6) (Figure 10).

### Association rule analysis

**Figure 11:**
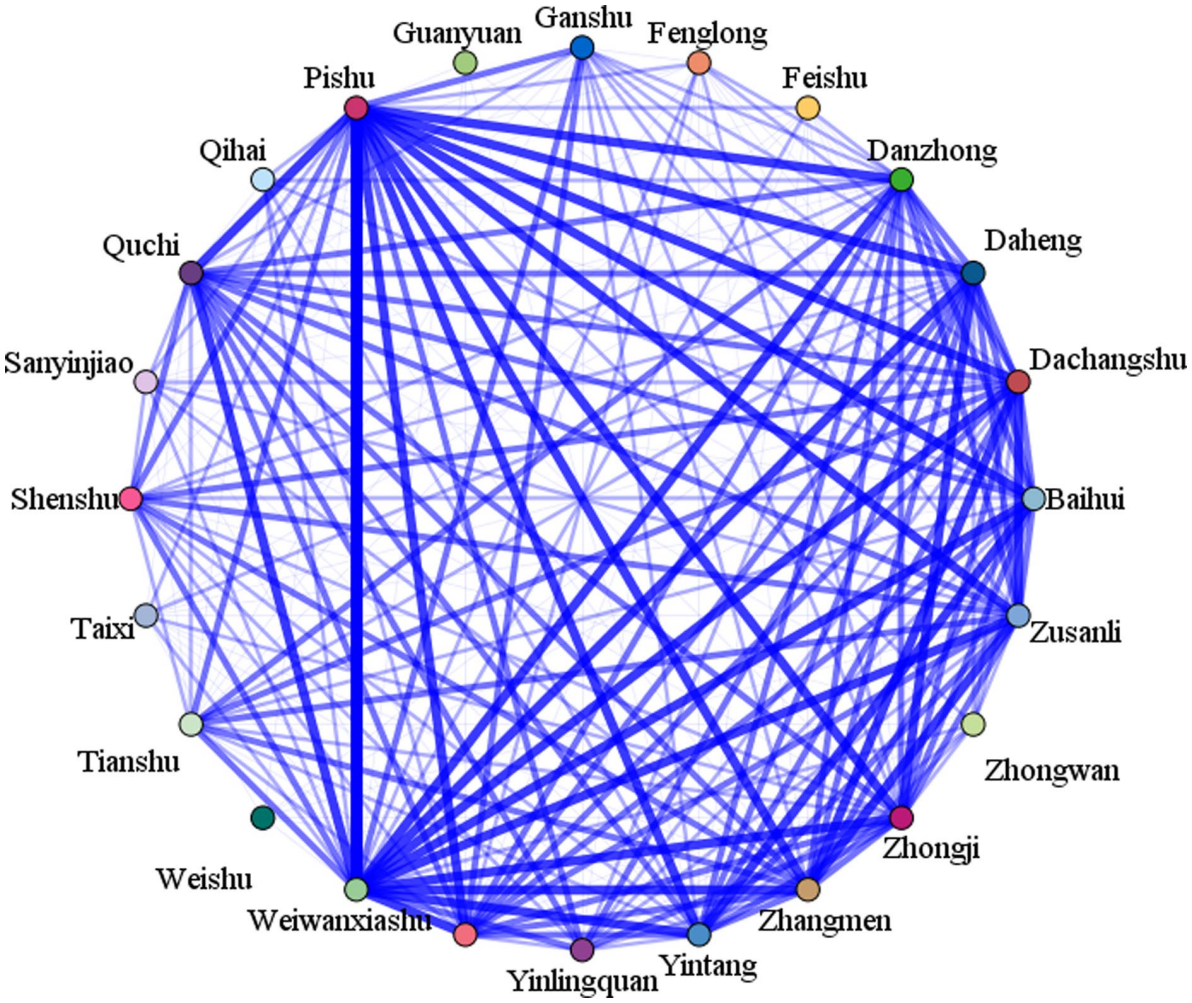
Association rule analysis.

When support ≥30%, confidence ≥100%, benefits ≥1.0, and the number of antecedent items was 6, a total of 6 schemes of acupoint combination were obtained, which all consisted of Pishu (BL20), Weiwanxiashu (EX-B3), Zusanli (ST36), Shenshu (BL23), Sanyinjiao (SP6), Weishu (BL21), and Taixi (KI3), as shown in Table 3. A mesh diagram of the association rules is shown in Figure 11.

**Table 3:** Analysis of association rules of core acupoints.

Preceding paragraph	Behind paragraph	Support/%	Confidence/%	Benefits
Weiwanxiashu	Taixi, Weishu, Sanyinjiao, Shenshu, Zusanli, Pishu	30.00	100.00	1.11
Pishu	Taixi, Weishu, Sanyinjiao, Shenshu, Zusanli, Weiwanxiashu	30.00	100.00	1.11
Zusanli	Taixi, Weishu, Sanyinjiao, Shenshu, Pishu, Weiwanxiashu	30.00	100.00	1.25
Shenshu	Taixi, Weishu, Sanyinjiao, Zusanli, Pishu, Weiwanxiashu	30.00	100.00	1.43
Sanyinjiao	Taixi, Weishu, Shenshu, Zusanli, Pishu, Weiwanxiashu	30.00	100.00	1.67
Weishu	Taixi, Sanyinjiao, Shenshu, Zusanli, Pishu, Weiwanxiashu	30.00	100.00	2.50
Taixi	Weishu, Sanyinjiao, Shenshu, Zusanli, Pishu, Weiwanxiashu	30.00	100.00	3.33

## Discussion

### Research background and significance

Diabetes is a major disease threatening human health and is the fifth leading cause of death worldwide (27). Pre-diabetes is a high-risk state between normal and diabetes, and the lifetime risk of progression from pre-diabetes to diabetes has been reported to be as high as 74% in people aged 45 years (28), making it important to block the progression of pre-diabetes to diabetes at an early stage (29). Although lifestyle interventions, such as dietary control and exercise, reduce the risk of conversion of pre-diabetes to diabetes, most patients have difficulty adhering to such habits, and lifestyle control alone does not prevent the development of isolated IFG to type 2 diabetes (30). The application of ACE in diabetes can be traced back to the 1990s, when Li et al. (31) discovered in 1992 that ACE has the potential to lower blood glucose levels. Subsequent experimental studies have confirmed that ACE can inhibit apoptosis of pancreatic β-cells, enhance insulin sensitivity, correct lipid metabolism disorders and improve vascular endothelial function in type 2 diabetic rats (32, 33). It was not until 2010 that Zhang et al. (34) found that ACE therapy could promote the reversal of pre-diabetes to normal glucose tolerance, thus reducing the incidence of diabetes, and the potential role of ACE for pre-diabetes has subsequently been emphasized by more and more researchers. In addition, clinical studies have confirmed that ACE effectively improves the prognosis of diabetic complications such as diabetic nephropathy, diabetic gastroparesis, and diabetic peripheral neuropathy (35–37), suggesting that ACE has the potential to treat diabetes and its complications. To our knowledge, this is the first meta-analysis and data mining of ACE for the treatment of pre-diabetes, aiming to assess the benefits and risks of ACE and investigate the optimal acupoint selection strategy for ACE in the treatment of pre-diabetes.

### Clinical efficacy and safety evaluation

Regarding the clinical effective rate and blood glucose endpoints, compared with conventional treatment, combined ACE and conventional treatment significantly increased the clinical effective rate by 51% and decreased HbA1c by 0.45%, FBG by 0.61 mmol/L, and 2 h PG by 0.77 mmol/L, suggesting that ACE was effective in lowering the blood glucose of pre-diabetes patients. For every 0.5 mmol/L increase in FBG in pre-diabetes patients, the risk of developing diabetes increases by 2–3 times (38), which implies that the blood glucose-lowering effect of ACE could help to reduce the risk of progression to diabetes in pre-diabetes patients.

Regarding the blood lipid endpoints, compared with the conventional treatment, combined ACE and conventional treatment significantly reduced TC by 0.37 mmol/L, TG by 0.49 mmol/L, and LDL-C by 0.23 mmol/L, and increased HDL-C by 0.16 mmol/L, suggesting that ACE has the additional benefit of modulating blood lipids. A study on Chinese adults showed that hyperlipidemia was associated with elevated blood glucose, and the prevalence of type 2 diabetes was significantly higher in patients with hyperlipidemia (39), implying that the effect of ACE in regulating blood lipids may be beneficial for further reducing the risk of diabetes development.

Regarding the bodyweight endpoint, although the combined ACE group had a lower BMI, it was not significantly different from that of the conventional treatment group. This result was unexpected, because many previous studies have confirmed the weight-reducing effects of ACE (40, 41). There are two possible explanation for this finding. First, the selection of acupoints differs in the treatment of pre-diabetes and obesity by ACE, and different selection schemes may lead to differences in the effects of ACE. Second, the BMI of the study only included a sample size of 588, and the negative results may have stemmed from an insufficient sample size. Therefore, it is not clear whether ACE reduces the BMI in pre-diabetes patients, although the results of this meta-analysis negate the benefit of ACE on BMI.

Adverse events in the combined ACE group were comparable to those in the conventional treatment group, suggesting that ACE does not increase adverse risks. Five adverse events related to ACE were reported in the included studies, including 2 cases of low-grade fever, 2 cases of subcutaneous hematoma, and 1 case of pain. Subcutaneous hematoma and pain are mainly related to the acupuncture needle and manipulation technique of embedding, which can generally be relieved on their own, whereas the appropriate selection of acupuncture needle and manipulation technique could help reduce the incidence of subcutaneous hematoma and pain (42). Low-grade fever is mainly related to the patient’s condition and is mostly an allergic reaction triggered by an acupuncture needle, and its overall incidence is low (42).

In the subgroup analysis of the frequency of embedding change, although both ≤15 days and > 15 days between embedding change had a beneficial effect on HbA1c, ≤15 days between embedding change reduced HbA1c by 0.60%, whereas >15 days of embedding reduced HbA1c by only 0.22%, suggesting that a higher frequency of embedding change may have greater benefit. In the subgroup analysis of treatment duration, a course of ACE for 12 weeks was effective in reducing HbA1c levels by 0.59%, whereas neither an 8-week course nor a 24-week course had a significant benefit on HbA1c levels. Only 60 patients were included in the analysis of the 8-week course, and only 156 patients were included in the analysis of the 24-week course; therefore, the negative results may be attributable to the limited sample size. In the subgroup analysis of drugs, ACE, with or without metformin, was associated with a significant reduction in HbA1c levels, suggesting that ACE has a broader scope of application.

### Analysis of treatment mechanisms

Pre-diabetes and type 2 diabetes share some similar mechanisms. Among these, IFG is mainly due to hepatic insulin resistance, with a reduced ability of glucose to stimulate its own uptake and inhibit its own production, resulting in elevated endogenous glucose levels (43). IGT is mainly characterized by skeletal muscle insulin resistance, which results in delayed glucose uptake and cellular dysfunction (44). Currently, there are few studies on the mechanisms of ACE in the treatment of pre-diabetes and diabetes, which include both aspects: in the first place, ACE can effectively inhibit the apoptosis of pancreatic islet β-cells in rats with type 2 diabetes, which is comparable to the effect of rosiglitazone by 0.2 mg/kg (32). This effect may be through inhibiting the activation of Pande and caspase-3 in pancreatic β-cells by hyperglycemia (44). In the second place, ACE reduces insulin resistance (45). First, ACE may enhance insulin sensitivity by improving vascular endothelial cell function, thereby lowering blood glucose (33). Second, ACE may reduce insulin resistance by increasing ghrelin (21). Third, ACE may enhance insulin sensitivity by lowering serum apelin and elevating serum glucagon-likepeptide-1 (GLP-1), subsequently lowering blood glucose levels (46).

### Data mining for acupoint selection schemes

The data mining results showed that Pishu (BL20), Weiwanxiashu (EX-B3), Zusanli (ST36), Shenshu (BL23), and Sanyinjiao (SP6) were the high-frequency acupoints for ACE in the treatment of pre-diabetes, whereas Pishu (BL20), Weiwanxiashu (EX-B3), Zusanli (ST36), Shenshu (BL23), Sanyinjiao (SP6), Weishu (BL21), and Taixi (KI3) together constituted the most commonly used ACE combination in the treatment of pre-diabetes. According to the traditional Chinese medicine theory, Pishu (BL20), Shenshu (BL23), and Weishu (BL21) belong to the bladder meridian, Taixi (KI3) belongs to the kidney meridian, Zusanli (ST36) belongs to the stomach meridian, Sanyinjiao (SP6) belongs to the spleen meridian, and Weiwanxiashu (EX-B3) is a non-meridian extra acupoint. Together, these acupoints play a role in invigorating the spleen and kidney, nourishing yin, and clearing heat, which corresponds to the pathogenic mechanism of pre-diabetes with deficiency of the spleen and kidney and dry heat-impairing fluid. Based on this result, we recommend Pishu (BL20), Weiwanxiashu (EX-B3), Zusanli (ST36), Shenshu (BL23), Sanyinjiao (SP6), Weishu (BL21), and Taixi (KI3) as the selection of acupoints in the treatment of pre-diabetes.

**Figure 12:**
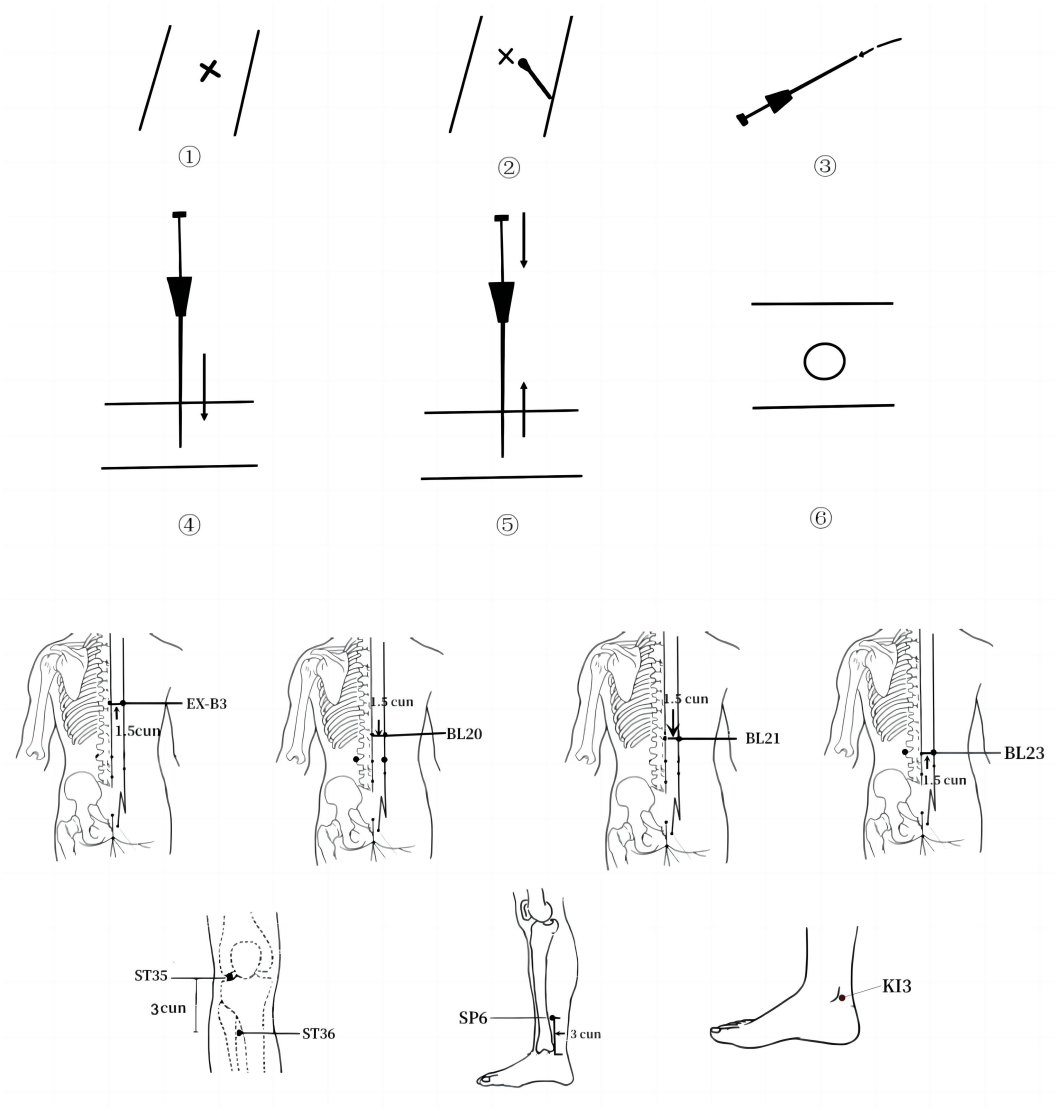
ACE flow diagram.

The specific operation method is as follows (Figure 12): (i) Select acupoints for positioning and mark them. (ii) Expose the skin of the target acupoint region and disinfect it. (iii) After disinfecting hands and donning sterile gloves, place the absorbable surgical suture into the front of the needle tube and attach the needle core to the back, angling it slightly so that the absorbable **s**urgical suture does not fall out. (iv) Insert the needle. Use one hand to fix the acupoint while using the other hand to insert the needle perpendicularly to the skin surface of the acupoint. When the depth of needle is in the range of 0.5–1 cm, apply lifting, inserting, and twisting to stimulate the acupoint and ask the patient if they feel pain, which is described as “Deqi.” (v) Withdraw the needle: After the patient feels Deqi, push the needle core and withdraw the needle tube, and the absorbable surgical suture in the muscle or subcutaneous tissue of the acupoint. (vi) After the needle is removed, press the puncture wound with a sterile dry cotton ball (swab) to stop bleeding, and then cover with a round plaster to prevent infection.

### Limitations and future studies

This study has some limitations. First, both the meta-analysis and data mining included only nine papers, which may have reduced the precision of the results. Second, all the studies were conducted in China, so the findings may not be applicable to people of other ethnicities. Third, the longest duration of treatment in the included studies was only 24 weeks; therefore, the results of the study reflect the short-term effects of ACE in the treatment of pre-diabetes, and the long-term effects of ACE in treating pre-diabetes patients are unclear. Fourth, the recommended selection scheme for ACE is based on the results of data mining, and its efficacy and safety need to be verified in multicenter clinical trials.

Further studies are required to address the limitations of existing studies. First, the recommended ACE scheme needs to be tested in a large multicenter clinical trial to investigate its efficacy and safety in the treatment of pre-diabetes. Second, the trial needs to be designed to enable subgroup analyses of the effects of ACE on pre-diabetes patients by sex, age, ethnicity, disease duration, baseline blood glucose, and baseline weight. Third, the follow-up period should be extended to 1 year or longer to investigate the long-term effects of ACE in pre-diabetes patients.

## Conclusion

ACE can effectively improve blood glucose and lipid levels in pre-diabetes patients with a favorable safety profile. The ACE treatment program consisting of Pishu (BL20), Weiwanxiashu (EX-B3), Zusanli (ST36), Shenshu (BL23), Sanyinjiao (SP6), Weishu (BL21), and Taixi (KI3) is expected to be a complementary scheme for the treatment of pre-diabetes.

## Data availability statement

The original contributions presented in the study are included in the article/Supplementary material, further inquiries can be directed to the corresponding author.

## Author contributions

YFY: Conceptualization, Supervision, Writing – original draft. XX: Methodology, Supervision, Writing – original draft. DT: Data curation, Methodology, Writing – original draft. YMY: Formal analysis, Methodology, Writing – original draft. XY: Formal analysis, Methodology, Writing – original draft. RY: Writing – review & editing.
