# Supplementary figures and images for: A study on the use of acupoint catgut embedding in the treatment of pre-diabetes: a meta-analysis and data mining approach
Source: Front Public Health. 2023 Dec 7;11:1282720. doi: 10.3389/fpubh.2023.1282720 (PMC10733528; doi:10.3389/fpubh.2023.1282720)

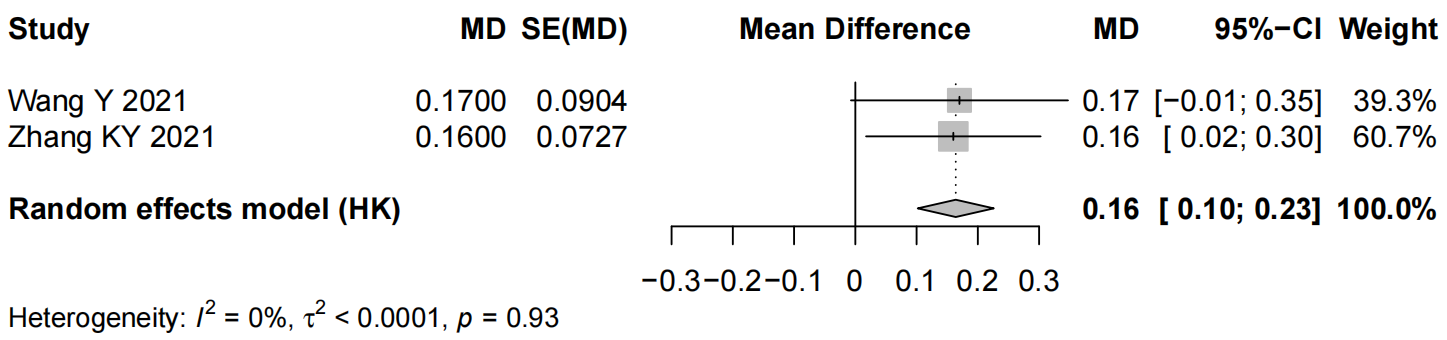

Supplement: Supplementary file 1 [file Image_1.JPEG]
